# Regional Planning for Health and Beauty—A Vision of a New South Yorkshire

**Published:** 1923-02

**Authors:** 


### REGIONAL PLANNING FOR HEALTH AND BEAUTY.

### A VISION OF A NEW SOUTH YORKSHIRE.

It has hitherto been regarded as inevitable that the growth of industrial enterprise should bring with it both squalid ugliness and unhealthy conditions, and hitherto industrial districts have been able to develop their natural wealth only by sacrificing their natural beauties. The town of Doncaster, magnicently situated on the Great North Road, and surrounded by much pleasant agricultural country, now sees before it a future of great promise as the coal measures to the east are increasingly opened up. But that yet another dreary wilderness may not be created, the local authorities, at the suggestion of the Minister of Health, have drawn up a Regional Planning Scheme—the first that has been prepared in this country—intended to show that "industrial prosperity can be achieved without loss of amenity or health," and the Committee has just issued its exceedingly valuable and illuminating report through the University Press of Liverpool and Messrs. Hodder & Stoughton (10s. net). The Joint Committee of the local authorities which, by the hands of Professor Patrick Abercombie, of Liverpool University, and Mr. T. H. Johnson, architect and surveyor, of Doncaster, has drawn up the report has done its work admirably. The document is complete and detailed, it leaves no aspect of the subject untouched, it is beautifully produced, and includes an appendix on coal subsidence. It is, moreover, admirably illustrated with maps and process blocks. Considerable sections of it are necessarily of purely local interest, but much of it is of general importance as breaking new ground. The report is, indeed, as a pioneer document, likely to be of historical importance as a foreshadowing of the great future of regional zoning.

### Grouping New Industries.

The Committee point out that man, in the haphazard way of the great but sad years when industrial development began to take its *essor*, and little as he recked it, prepared the way for regional planning in this district :—

"The Doncaster region equipped by man, without any forethought of its industrial future, with unrivalled roads and railways, possesses in its most difficult feature, the low-lying tracts, the opportunity of grouping new industries in a manner at once fortunate for themselves and for those who will work in them. Coal, the *primum mobile* of this growth, and the cause of its attendant difficulties, is found at such a depth that the worst infirmity of a mining district—surface cracks and frequent pit-heads—is absent. But it is in the grouping of the population that the chief attractiveness of the picture is found. It is no longer, one hopes, possible for a single city of the numbers and density of Leeds or Sheffield to come into existence ; and one contemplates with only less horror a town, equal in population, but with its people spread out at the rate of fifty to the acre."

### The Ideal Community.

The Committee see a more pleasing vision than this—a vision in which the fairness of Nature shall not be marred by the vileness of man :—

"Instead, there should spring up in this region ten or more communities—new, or so changed as to rank as new—towns complete in every respect, but of moderate size, manageable in their loose texture. Central to these, but in no sense dominating their individual existence, is to be a city, neither swollen nor tentacular, but in the truest meaning of the word metropolitan. For major pleasures, for higher studies, for contact with great art . . . the inhabitants of the surrounding communities would have, within easy reach, this focus of civilisation. Agricultural land, small holdings, allotments, and ample space for playing fields would form the natural matrix to these human and industrial aggregates, cementing them together, and at the same time keeping them apart. Social life in such a region should be rich and varied ; there need be no tame village existence in the smaller communities, nor less of touch with Nature in the central city."

### Health and Contented Labour.

The area with which this report is concerned extends for several miles around Doncaster and includes about fifty villages. The restrictions upon an unfettered use of the land within it are naturally only of a general nature :—

"It is clear that the low-lying land is not so healthy for residential purposes as the higher ground ; and, where the former has been lowered so as to make even land drainage a difficulty, its unsuitability for an increased population becomes manifest. At the same time, the pit-heads of several collieries are situated on this low ground, and more might be opened on it. Again, the proximity of waterways and railways suggest that the example of Messrs. Pilkington at Kirk Sandall will be followed ; and furthermore it is necessary to the prosperity of the district that collieries should flourish and further factories be established. Labour is essential to development, but it must also be healthy and contented labour. It would never do to perpetuate the mistake of Sheffield in the past, in allowing houses to be built in the lower Don Valley, where they are unhealthy in themselves and occupy land which should be available for works. The right solution of the problem, accordingly, would be to prohibit the erection of more houses in those villages which are situated below a certain datum line and to stop the establishment of new housing schemes on the same low land, while at the same time by means of carefully considered local transport to provide facilities to render sites on the higher ground readily accessible from existing or new collieries or factories upon the lower."

### Parks and Playgrounds.

In an area only the fair fringe of which has been touched by industrialism, it has been impossible at this early stage to select large tracts of land and earmark them for parks and playgrounds :—

"It is very necessary that this systematic provision of open spaces should be borne in mind, so that the large tracts already possessed by the Doncaster Corporation and the local spaces to be acquired by the new communities may be knit into a logical regional park system. The advantages of co-operation are perhaps nowhere more obvious than in the joint preparation of a scheme for open spaces and the joint administration of them."

### Preserving Natural Beauties.

With the exception of Sprotborough, where the outcrop of magnesian limestone has been cut through by the Don forming a gorge of extreme beauty, "there are no natural features which by reason of their beauty on the one hand, and unsuitability to other forms of use on the other, renders them manifestly suitable for preservation." The fine ruin of Conisbrough Castle is already well looked after, and the other historic features of the neighbourhood most worthy of preservation are some of the old villages —Campsall, Burghwallis, Hickleton, High Melton, Marr, and Hooton Pagnell:—

"Wherever possible, when these places are found on important traffic routes, the main stream of traffic should be carried beside them by means of a bye-pass; the old method of widening the village street was both costly and destructive of its charm. . . . For a growing district such as this, it is impossible to over-estimate the value of the preservation of its beautiful relics of the past. Mention should also be made of some of the fine tree-planted avenues in the region; the Great North Road where it enters Doncaster, Steep Bridge Lane, near Rossington, and others; no road widening should be allowed to interfere with those trees, which could be worked into the new width where required. It is to be regretted that the most durable type of tree has not always been planted in the past, some of them of an age of 80 years being badly decayed. . . . The tree planting on all wide new roads should be most carefully studied."

### Doncaster as "A City of the First Magnitude."

The town of Doncaster itself will form a handsome and dignified core and capital for this new region:—

"Doncaster has the makings of a fine city of the first magnitude; the approach to its main street—at any rate from the south-east along the Great North Road—is perhaps as fine as the entrance to any town, great or small, in the United Kingdom. The ownership of a great corporate estate, both in the town and around it, gives it opportunities for carrying out projects and reaping the financial benefits of prosperity rare in this country. Its architectural character, not only in the Parish Church and famous Mansion House, but in many dignified houses dating from the eighteenth century, has nothing provincial about it; though one deplores the destruction of many fine old buildings which would have added historic interest to its streets. Its road plan, again, is no muddle, like that of many larger towns, but, thanks to a level site and a Roman origin, is simple and easily grasped, and, one might add, easily capable of expansion."

### Its Future.

Nor will the scheme involve any diminution of its attractions, while its importance will be vastly increased:—

"It need not be thought that Doncaster will suffer in consequence. She will on the contrary react and ever increase in the surrounding communities; an exchange having a similar relation to them that the Manchester Exchange does to the surrounding county boroughs will probably come into existence; her shops will become great central stores and emporiums; in a word, she will become the financial heart of the Region. . . . Just as the Region is zoned in large tracts, so it is desirable to lay down a zoning scheme for the City of Doncaster, in order that houses and factories may not be jumbled together, and that business areas may be encouraged where appropriate. . . . Often spaces should be provided on a systematic basis, from the small children's playground, at frequent intervals, to the large local parks, so that every part of the town may be adequately served. Access to these should, so far as possible, partake of the nature of tree-planted avenues or parkways in contrast to hot or arid streets and dusty motor routes."

The report deserves the careful attention of every local authority in the country, and of every person who prefers order to chaos.

---

A Healthy Occupation.—It would seem that the making of appliances intended to restore health is in itself a healthy occupation. The well-known firm of Messrs. Maw & Sons, surgical instrument makers, have a list of twenty-two employés who have been with them for forty years or more, and one of them, John Denton, who has now retired, never missed one day during a service of sixty-two years. He was never ill and never late.

